# The Design Challenges for Dog Ownership and Dog Walking in Dense Urban Areas: The Case of Japan

**DOI:** 10.3389/fpubh.2022.904122

**Published:** 2022-04-29

**Authors:** Mohammad Javad Koohsari, Akitomo Yasunaga, Gavin R. McCormack, Tomoki Nakaya, Yukari Nagai, Koichiro Oka

**Affiliations:** ^1^Faculty of Sport Sciences, Waseda University, Tokorozawa, Japan; ^2^Faculty of Liberal Arts and Sciences, Bunka Gakuen University, Tokyo, Japan; ^3^Department of Community Health Sciences, Cumming School of Medicine, University of Calgary, Calgary, AB, Canada; ^4^Faculty of Kinesiology, University of Calgary, Calgary, AB, Canada; ^5^School of Architecture, Planning and Landscape, University of Calgary, Calgary, AB, Canada; ^6^Graduate School of Environmental Studies, Tohoku University, Sendai, Japan; ^7^School of Knowledge Science, Japan Advanced Institute of Science and Technology, Nomi, Japan

**Keywords:** pets, cities, urban design, Asia, high density, public health, healthy aging, evidence-based design

## Abstract

There has been growing interest in the role of pet ownership, particularly dog ownership, in managing noncommunicable diseases. The built environment can act as a facilitator or barrier to owning a dog or dog walking. Nevertheless, limited studies conducted in different geographical contexts have examined how the built environment can influence dog ownership and dog walking. In this interdisciplinary article, using Japan as a case study, we identify key design challenges to owning and walking dogs in dense urban areas as a means of promoting health and wellbeing.

## Introduction

The prevalence of noncommunicable diseases, such as cancer, diabetes, mental illness, and cardiovascular diseases, is among the top global health concerns, responsible for 71% of deaths globally (1). More than half of these deaths occur in Asia, a place with an estimated population of 4.7 billion (59.8% of the total world population) (2, 3). For instance, in the Western Pacific region, such as Japan, China, Australia, Republic of Korea, and the Philippines, noncommunicable diseases are the leading causes of death and disability, accounting for 80% of all deaths (4). Hence, public health initiatives and organizations have focused on monitoring modifiable risk factors, such as physical inactivity, unhealthy diet, alcohol consumption, and smoking, to prevent noncommunicable diseases.

There has been growing interest in the role of pet ownership, particularly dog ownership, in preventing noncommunicable diseases (5–10). Findings from a systematic review and meta-analysis showed that compared with nonowners, dog owners had a 31% risk reduction for cardiovascular mortality (5). Several studies have demonstrated the positive effects of dog ownership on lowering noncommunicable disease risk factors, such as physical inactivity (11–13), hypertension (14), and mental illness (15). For instance, a study conducted in the UK found that dog owners were four times more likely to meet the physical activity guideline (moderate to vigorous intensity physical activity >150 min per week) than nonowners (11). Another recent study conducted in Japan found that dog ownership was negatively associated with objectively assessed sedentary time, an independent health risk, in adults (12).

Despite the health benefits, owning and caring for dogs can be challenging, especially for those residing in urban areas. While most of the health benefits of dog ownership are derived from dog walking, not all owners walk their dogs (16–19). A study in the USA observed that 30% of dog owners had not walked their dogs in the past week (18). Another study undertaken in Japan found that 44% of dog owners did not report walking their dogs (16). Socioecological models of health suggest that multiple factors, such as personal, social, and built environment factors, can influence people's decisions to engage in healthy behavior (20). These models can also be adapted in relation to dog ownership and dog walking (21, 22). There has been a recent emphasis on the built environment's role in dog ownership and dog walking (8). The built environment can be described as “the human-made space in which people live, work, and recreate on a day-to-day basis” (23). Several aspects of the built environment are identified as correlates of dog ownership and dog walking (17, 24–26). For instance, access to dog-supportive parks was associated with dog walking in a sample of adults in Australia (24). In a Canadian sample, dog walking was associated with connected street networks and nearby off-leash greenspaces (25).

Nevertheless, limited studies conducted in different geographical contexts have examined how the built environment can influence dog ownership and dog walking. Most of the existing evidence comes from sprawled lower density areas in Western countries, such as Australia (24, 27, 28), Canada (17, 25), the UK (29), and the USA (26). Notably, little empirical evidence on dog-built environment relations exists in the context of dense urban areas in Asia (30, 31). There has been a dramatic rise in urbanization globally, and it is estimated that two-thirds of the world population will reside in urban areas by 2050 (32). Asia has one of the highest urbanization rates and hosts most of the world's high-density megacities (with a minimum population of 10 million) (33). High-density cities are also not necessarily a feature of Asia alone, as they exist in Europe (i.e., London) and the USA (i.e., New York City). Investigating how living in these dense urban areas affects dog ownership and dog walking can provide lessons for other highly populated regions where urbanization and densification are increasing.

Following the trend on dog-built environment relations, in this interdisciplinary article, we have identified key design challenges that impact owning and walking dogs as a means of health and wellbeing promotion in dense urban areas using Japan as a case study.

## Dog Ownership and Dog Walking in Japanese Cities

Dogs have lived with people since ancient times as domestic animals. In Japan, dogs were noted in ancient history books, such as *Nihon Shoki*, the oldest extant official history book in Japan, which was completed around 720 AD. In Japan, a law on rabies prevention was enacted in 1950, requiring all dogs to be registered with local authorities and vaccinated against rabies. Except for some occupational dogs (e.g., police dogs), all dogs must be registered by their owners.

According to Japan's Ministry of Health, Labor, and Welfare, the number of registered dogs nationwide in 2020 was 6,090,244 (4,828 per 100,000 population) (34). This figure is lower for urban areas. In the top two most densely populated prefectures in Japan, Tokyo and Osaka, the numbers of registered dogs are 510,511 (3,634 per 100,000 population) and 383,030 (4,334 per 100,000 population), respectively. These figures are slightly lower in the prefectural capitals (principal cities): 3,322 per 100,000 population in the 23 wards of Tokyo and 3,312 per 100,000 population in Osaka City.

Japan is currently a super-aged society with one of the world's lowest fertility rates (35). In this context, dogs are viewed as important for supporting health and wellbeing. For example, a 2010 national survey on pets reported that owning a pet (the highest number of respondents said they had a dog) provided mental benefits, such as offering enrichment and comfort in life, supporting a more peaceful home, improving children's minds, and being fun to raise (36). According to the Japan Pet Food Association, 11.7% of people aged in their 60's and 9.2% of people in their 70's owned a dog in 2021 (37). As the aging population increases, dog ownership may provide a valuable opportunity to enhance the quality of life, especially among the elderly in Japan. Consistent with the rapid decline in Japan's population, urban shrinkage will continue. The vacant lands and houses resulting from this shrinkage can provide an opportunity to (re)design dog-friendly environments.

Several studies have investigated the effects of dog acquisition on transmitting infections such as Giardia cysts, Strongyloides, and Echinococcus in Japan (38–40). For example, a study that obtained data from many regions in Japan found Giardia cysts in 10.9% of dogs (out of 2218 dogs). However, they concluded that the chance of human infection from dogs was low (38). Another study conducted in the Amami Islands, Japan, examined the cross-infection of Strongyloides between residents (*n* = 660) and their dogs (*n* = 55). They found that 10% of dogs had Strongyloides infection. However, no evidence of cross-infection between humans and dogs was found in their sample (39). Although evidence from other countries has also suggested that animal-to-human transmission of these infections may be uncommon (22, 41), proper dog pet management for zoonotic disease prevention is necessary, especially in highly populated areas.

## The Design Challenges for Dog Ownership and Dog Walking in Japan's Urban Areas

### Buildings

As the primary setting of daily life, buildings may act as facilitators or barriers to dog owners and dog walking (8, 22). Notably, the size of housing units is a key design element that could impact residents' behaviors and wellbeing (42, 43). According to Japan's 2018 Housing and Land Survey, the average size of a housing unit (excluding yards) in Japan was 92.06 square meters (44). This figure is smaller in major urban areas. For instance, Tokyo, the capital of Japan and the most densely populated prefecture, has the smallest average housing unit size among all Japanese prefectures, with 65.18 square meters. Similarly, the average sizes of a housing unit in Osaka (the second most densely populated prefecture in Japan) and Kanagawa (the third most densely populated prefecture in Japan) are minimal, with 76.20 square meters and 77.80 square meters, respectively. In addition, the percentages of owner-occupied houses in Tokyo (45.0%), Osaka (54.7%), and Kanagawa (59.1%) are lower than the Japanese national average (61.2%) (44). This shows that many people live in rented housing such as condominiums and flats. The relatively small living spaces in dense urban areas and the typical absence of private yards could limit people from keeping dogs in their homes. Small living spaces could also limit the breeds of dogs that can be owned, with preferences for smaller breeds that typically have lower exercise requirements. Outdoor design features of buildings may also affect dog walking. For example, hooks for leashing the dog outdoors when the owner enters a building are helpful for dog owners who wish to walk their dogs while traveling to destinations (e.g., supermarkets, cafes). Such outdoor design features may be even more essential in dense urban areas where small housing units are prevalent. Like many other cities, these dog-friendly outdoor design features of buildings are mostly missing in Japan's urban areas.

There are also several rules and norms related to buildings that impact dog ownership and dog walking. Finding affordable and comfortable housing that permits dogs is a challenge in these areas' competitive housing rental market. Since many owners prefer not to rent their properties to dog-owners (to prevent possible damage to their property or disturbing neighbors), the rental price of such accommodation is higher in most cases. Such apartments may not also be located within convenient distances from popular destinations. Therefore, dog-permitted housing costs more and may force families with dogs, especially lower-income households, to live in areas where fulfilling daily activities is challenging. Even owning an apartment unit does not guarantee that a family can bring a dog to live there. Many urban apartments in Japan have “no-pet” rules for owners and renters. For instance, according to a major real estate company website in Japan, only 18.9 per cent (39,456 out of 209,290) of rental houses (flats, condominiums and houses) in the 23 wards of Tokyo allow pets (45). Additionally, although there have been some recent changes, most governmental offices, shops, and cafes still have strict rules for permitting dogs in urban areas in Japan. For example, most government city offices do ask dog owners to put their dogs in portable pet cages before entering the buildings, except for guided dogs. They also need to leave the premises if their dogs cause a disturbance to other visitors, such as barking or whimpering. Affordable facilities such as pet hotels and pet care centers in these dense areas may encourage individuals (especially singles) to own dogs.

### Public Open Spaces

**Figure 1:**
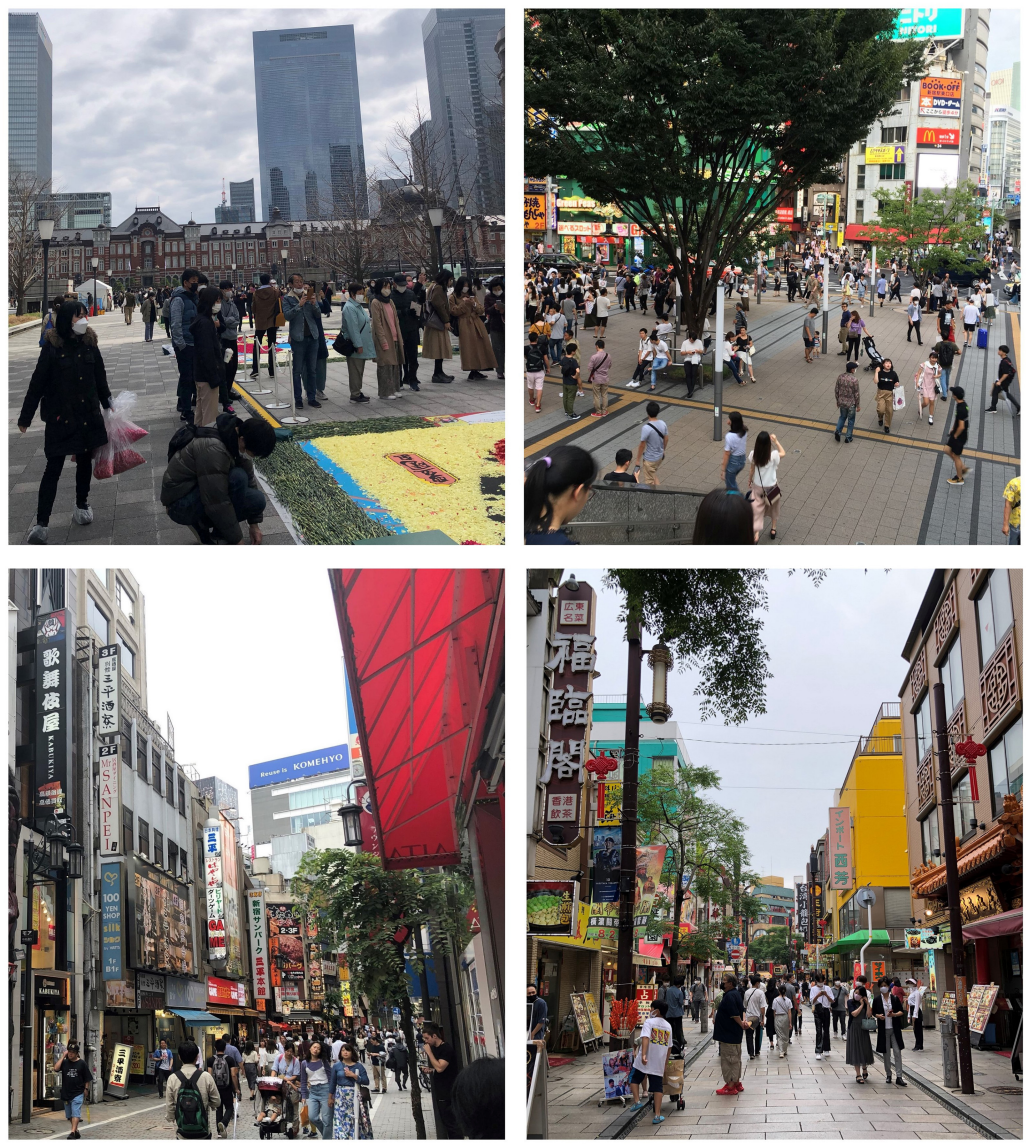
Overcrowding in public open spaces in Japanese urban areas (Source: the authors).

Public open spaces, such as streets, parks, and playgrounds, are vital neighborhood settings for being physically active. Several studies have shown the positive effects of public open spaces on dog walking (24, 25). A study conducted in Australia found that access to a dog-supportive park was associated with regular dog walking (24). Another study in Canada found that dog walking was positively associated with better access to a park with dog off-leash areas (25). In dense urban areas, where most housing units have no yard (or have a small yard), public open spaces may play an even more critical role in acquiring and walking a dog. However, several issues exist with public open spaces in this context that can negatively affect dog ownership and dog walking. Overcrowding of public open spaces is typical for Japan's urban areas and several other high-density Asian cities (Figure 1). In line with its definition in housing research (46), overcrowding in public open spaces can be defined as having too many people in a place to permit comfortable and appropriate physical and social interactions. The average density of people in Japanese urban areas is higher than that in the USA, Canadian, and Australian cities. For example, according to the national census in 2020, the population densities of Tokyo, Osaka, and Kanagawa are 6,402.6, 4,638.4, and 3,823.2 persons per square kilometer, respectively (47). In contrast, the population density of the densest capital in Australia, Melbourne, is 450 people per square kilometer (48). The overcrowding of public open spaces may discourage dog owners from walking their dogs frequently. There is potential for more conflicts between dog walkers and non-dog walkers in public open spaces (e.g., on crowded sidewalks and squares) in dense urban areas. This situation is echoed by the popularity of small breeds of dogs in Japan in the last decade. Some dog owners find such breeds attractive because they seem suitable for living in a small apartment in a neighborhood with limited public open spaces.

**Figure 2:**
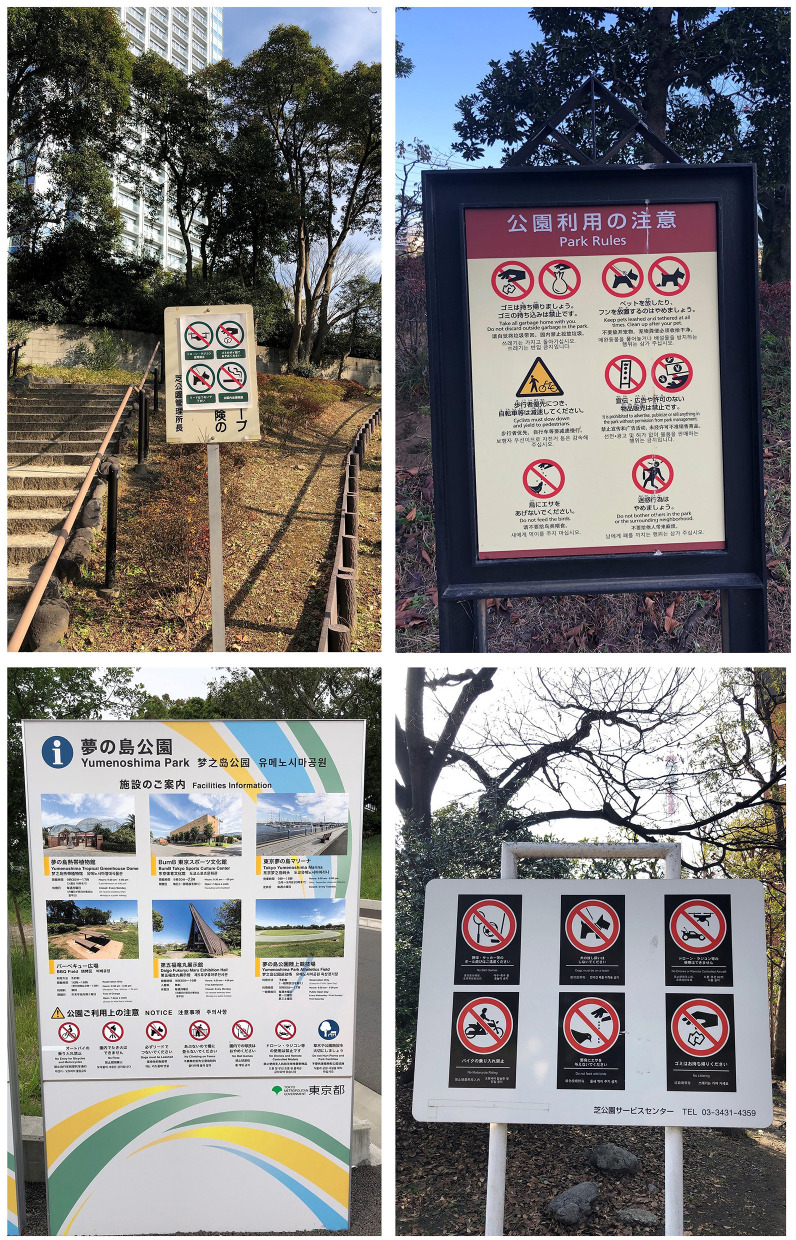
Lack of parks allowing dogs off-leash in Japanese urban areas (Source: the authors).

There is a noticeable lack of public green spaces in Japan's dense urban areas. Japan's top three highly populated prefectures, Tokyo, Osaka, and Kanagawa, had an average of only 7.4, 6.2, and 7.3 square meters of park space per person in 2019, respectively (49). In particular, the central city of each prefecture has even a smaller park space per person: 2.9 square meters in the 23 wards of Tokyo, 3.5 square meters in Osaka city, and 5.0 square meters in Yokohama city. In comparison with other large cities globally, these are not remarkable figures. For instance, Melbourne and Toronto have 116 and 28 square meters of green space per person, respectively (50, 51). In Japan, residential lands comprise only four per cent of national land because approximately three-quarters of Japan's national land is mountains (52). Thus, the lack of space limits the allocation and construction of new parks and green spaces within urban areas. Notably, Japan's urban parks and playgrounds are mostly well managed by local municipalities and organizations. However, these organizations usually set a long list of “to do” and “not to do” items for park and playground users. Not only can dogs not be off-leash in most of these parks, but the “no-pet” rule exists in some cases (Figure 2). While dense urban areas have some public dog-designated areas, their number and distribution cannot satisfy the needs of dog owners. Some dog-designated playgrounds have been built recently by private companies. However, their cost may discourage owners who want to use such facilities regularly.

Dog amenities, such as garbage disposal devices for dog waste, dog waste bags, and drinking water for dogs, support dog walking (41). These dog amenities are mainly absent in Japan's urban areas, as in most Asian cities. Additionally, most sidewalks in these areas are not physically separated from the vehicle flow. The lack of separated sidewalks reduces safety perceptions which eventually discourage dog walking (22). Nevertheless, having sidewalks to walk on is better than having no sidewalks for dog owners, even if such sidewalks are not physically separated from vehicle traffic or are crowded.

### Mobility-Related Features

There are some unique mobility-related features in Japan's dense urban areas that may be barriers to owning and walking dogs. The limited residential land and the organic (unplanned) nature of most Japanese cities have led to narrow sidewalks and streets. The lack of separated sidewalks and overcrowding can cause people to feel unsafe and uncomfortable, which discourages walking. This situation is even more problematic if dog owners want to walk their dogs on these busy sidewalks.

The high cost of car parking spaces is also a common experience in Japan's dense urban areas. This limits dog owners from visiting dog designated areas even if they own a car (and if there are such dog designated areas). There are also several rules in using public transport (buses and trains) for dog owners that limit them. Only small dog breeds, held in a travel crate, are permitted to ride trains and buses in most of Japan's urban areas. For instance, according to the East Japan Railway Company, a major railway company in Japan, small dogs may be carried on board for a fee as long as they are kept in a travel crate no more than 70 cm long and with a total length, width and height of no more than ~90 cm (53). Keio Dentetsu Bus, one of Tokyo's larger bus companies, also requires that pets be held in a basket or case with their door (lid) closed and that they not bother other passengers to be allowed to board buses (54).

**Figure 3:**
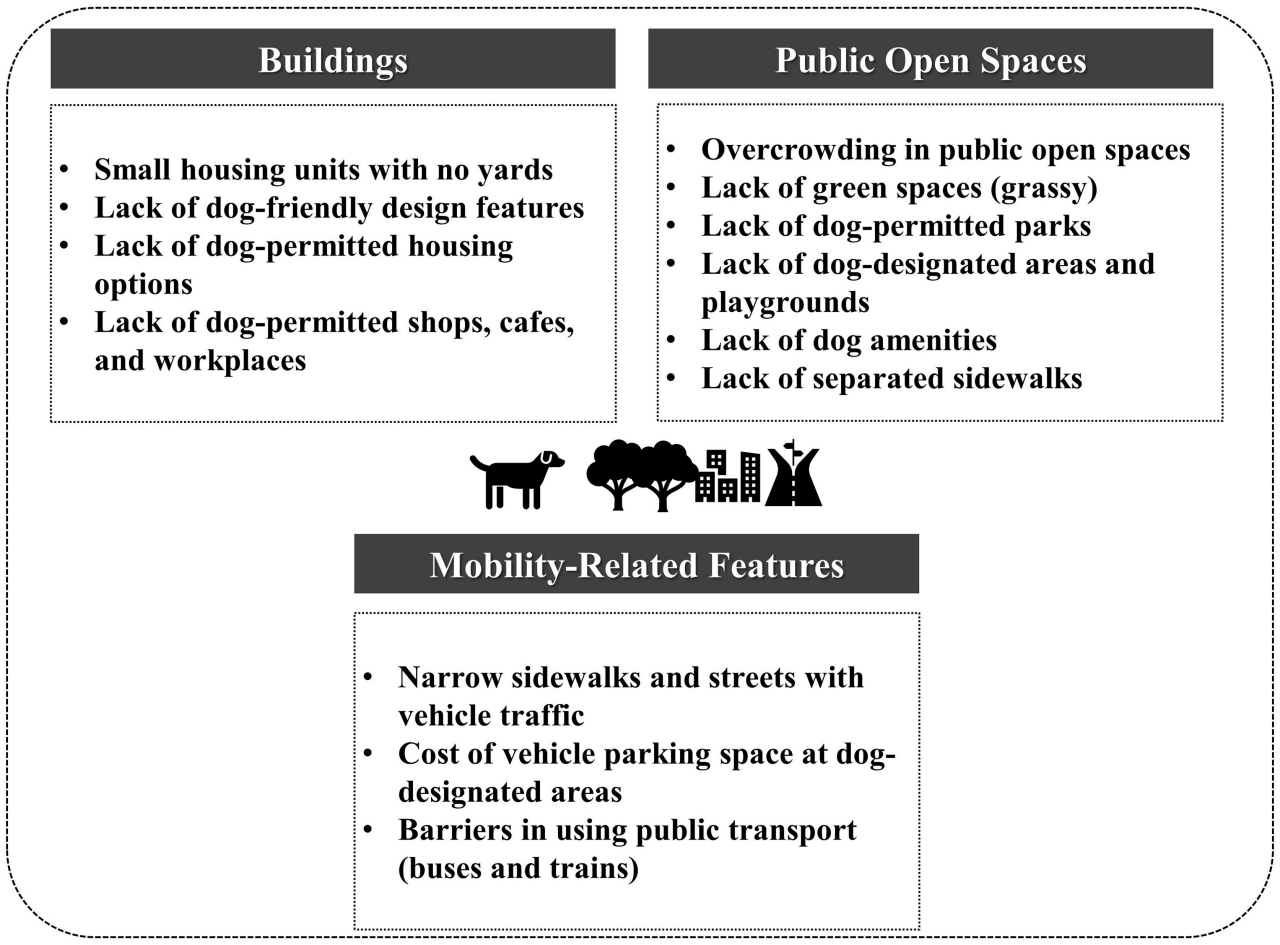
The design challenges of dog ownership and dog walking in dense urban areas: the case of Japan.

As we discussed above, there are several design challenges in Japan's dense urban areas in relation to dog ownership and dog walking. We have categorized these challenges into three physical built environment domains: buildings, public open spaces, and mobility-related features (Figure 3). To summarize, the design issues need to be explored in these three categories to provide evidence for promoting dog ownership and dog walking in dense urban areas. The effects of the small housing area and lack of dog-permitted spaces on dog ownership and dog walking need to be investigated in the buildings category. In the public open spaces category, overcrowding and lack of green spaces are common issues in dense urban areas. Barriers to using public transport and narrow sidewalks are mobility-related features in relation to owning and walking dogs. Nevertheless, there is a dearth of empirical studies on these topics in dense urban areas. Without such evidence, any design recommendations to improve the built environment for supporting dog ownership and dog walking remain somewhat speculative. Further studies conducted in dense urban areas are necessary to shed light on this topic.

## Conclusions

Dog ownership and dog walking have been found to promote physical and mental health in several ways. The design of the surrounding built environment can facilitate or hinder the expected health benefits of owning a dog. This article provides a critical perspective on the design challenges of dog ownership and dog walking in dense urban areas using Japan as a case study. Context-specific evidence on built environment-dog relations is needed to develop effective design solutions.

## Data Availability Statement

The original contributions presented in the study are included in the article/supplementary material, further inquiries can be directed to the corresponding author.

## Author Contributions

All authors listed have made a substantial, direct, and intellectual contribution to the work and approved it for publication.

## Funding

TN was supported by the JSPS KAKENHI (#20H00040). GRM is supported by a Canadian Institutes of Health Research Foundations Scheme Grant (FDN-154331). KO is supported by a Grant-in-Aid for Scientific Research (No. 20H04113) from the Japan Society for the Promotion of Science.

## Conflict of Interest

The authors declare that the research was conducted in the absence of any commercial or financial relationships that could be construed as a potential conflict of interest.

## Publisher's Note

All claims expressed in this article are solely those of the authors and do not necessarily represent those of their affiliated organizations, or those of the publisher, the editors and the reviewers. Any product that may be evaluated in this article, or claim that may be made by its manufacturer, is not guaranteed or endorsed by the publisher.
